# Promoter *P*_*PSP*1–5_-*BnPSP-1* From Ramie (*Boehmeria nivea* L. Gaud.) Can Drive Phloem-Specific GUS Expression in *Arabidopsis thaliana*

**DOI:** 10.3389/fgene.2020.553265

**Published:** 2020-12-16

**Authors:** Yunhe Wang, Yaning Bao, Yancheng Zheng, Ping’an Guo, Dingxiang Peng, Bo Wang

**Affiliations:** ^1^MOA Key Laboratory of Crop Ecophysiology and Farming System in the Middle Reaches of the Yangtze River, College of Plant Science and Technology, Huazhong Agricultural University, Wuhan, China; ^2^College of Tobacco Science, University of Guizhou, Guiyang, China; ^3^Hubei Key Laboratory of Edible Wild Plants Conservation and Utilization, University of Hubei Normal, Huangshi, China

**Keywords:** tissue-specific, promoter, UFW cloning, GUS expression, ramie [*Boehmeria nivea* (L.) Gaud.]

## Abstract

Isolation of phloem-specific promoters is one of the basic conditions for improving the fiber development and resistance of ramie phloem using genetic engineering. In this study, we isolated a ramie endogenous promoter (named *P_*PSP1*_-BnPSP-1*) and analyzed the function of its truncated fragments in Arabidopsis. The results show that *P_*PSP1*_-BnPSP-1* can drive the GUS reporter gene to be specifically expressed in the veins of Arabidopsis. After hormone and simulated drought treatment of the independent Arabidopsis lines carrying *P_*PSP1*_-BnPSP-1* and its truncated fragments, only *P_*PSP*1–5_-BnPSP-1* (−600 to −1 bp region of *P_*PSP1*_-BnPSP-1*) is stably expressed and exhibits phloem specificity. Our findings suggest that *P_*PSP*1–5_-BnPSP-1* can be used as a phloem specific promoter for further research.

## Introduction

Promoters play a vital role in the initiation and regulation of gene transcription. Constitutive promoters have been used earlier and widely in genetic engineering, and have played an important role in plant disease resistance engineering. However, it has gradually been confirmed that there are unavoidable defects behind their high expression-poor temporal and spatial specificity, which can cause foreign sources Gene silencing (Charrier et al., 2000) increases plant energy consumption and reduces biomass, which affects plant yield (Krishna et al., 2016), and the appearance of additional disease symptoms reduces plant resistance (Quan et al., 2018). The specific expression promoters that are gradually isolated and verified to meet the target requirements can alleviate such problems to a certain extent.

Bast fiber crops are the most productive main fiber plants among the natural fiber sources, and their yield and quality are directly affected by the growth and development of sclerenchymatous fibers in the plant phloem (Pari et al., 2015). The expression of genes related to phloem development is inseparable from the regulation of upstream promoters and developing endogenous phloem-specific promoters from plants has significant application prospects in improving the developmental characters of phloem fibers. Since the first maize sucrose synthase-1 (SH1) promoter was isolated and proved to drive the specific expression of the GUS fusion gene in tobacco phloem (Yang and Russell, 1990), more and more phloem-specific expression promoters were identified and verified from plants. In recent years, more studies have applied them to improve plant resistance and other aspects. Scholars constructed a binary vector with an Arabidopsis sucrose-H homologous gene (AtSUC2) promoter expression cassette to target phloem-specific expression, driving the reporter gene to be specific in the phloem of the leaf, petiole, and root of the transgenic plant Expressed, and the transgenic plants showed resistance to plant protozoa (mycoplasma-like prokaryotes) (Zhao et al., 2004). The study in tomato cytosolic fructokinase FRK1 suggest that SlFRK1 is involved in vascular tissue development and hydraulic conductivity in tomato plants and that SlFRK1 is important for normal phloem fiber development, together with SlFRK2 (Stein et al., 2017). Research on the identification of the phloem-specific activity of the poplar PtrDP3 promoter found that the transgenic Arabidopsis plants are not affected by abiotic stress or exogenously applied plant hormones (Nguyen et al., 2017). This study provides evidence of a strong phloem-specific promoter that is suitable for phloem-specific biotechnological modifications in plants. Related research on the promoter of the poplar tissue-specific gene xylosyltransferase gene provides a tool for changing the content of xylan covering the cellulose matrix (Porth et al., 2018). The development and verification of multiple uses makes the application of phloem-specific promoters possible.

Ramie has been used as a fiber crop in the world for more than 6,000 years. The fabrics made from its fiber are light and breathable, occupying an important position in the textile industry (Paiva Junior et al., 2004). However, disadvantages such as poor crease-resistance and difficulty in dyeing restrain the utility of ramie fiber (Sun, 2004). The directional improvement of ramie fiber quality by combining phloem-specific (preferentially expressed) promoters with fiber development-related genes is a promising approach. The endogenous promoters of ramie that have been reported so far include *mannanase* gene promoter (Ma et al., 2018), *phloem protein 2* (*PP2*) gene promoter (Guo et al., 2018), *germacrene D synthase* promoter, shoot apical meristem-specific regulator promoter (Guo et al., 2019a), and *sucrose synthase 1* (*BnSUS1*) gene promoter (Guo et al., 2019b), but *BnSUS1* is the only one that drives specific expression in vascular tissues. There are many ways to clone flanking sequences, and we isolated promoters via Universal Fast Walking method (UFW) (Myrick and Gelbart, 2002). Compared with Y-connector and TAIL-polymerase chain reaction (TAIL-PCR), the UFW method is characterized by strong specificity and low costs (Chen et al., 2015). Guo et al. (2018, 2019b) used this method to successfully obtain the promoter sequences of ramie *SUS1*, *PP2*, germacrene D synthase, and shoot apical meristem-specific regulator.

In the present study, we explored and cloned three genes which are specifically or predominantly expressed in the phloem of ramie, then fused GUS and transformed into Arabidopsis to determine the expression site and the expression level of GUS reporter genes. Moreover, the position of internal regulatory elements of the promoters and their roles in different treatments were studied, which providing a basis for subsequent molecular biological studies on ramie, a tool for ramie molecular breeding and new tissue-specific promoters for genetic engineering.

## Materials and Methods

### Experimental Materials

Ramie cultivar Huazhu No. 5 was obtained from the Ramie Germplasm Nursery in Huazhong Agricultural University. Wild-type Arabidopsis (Columbia ecotype) lines were used for experimentation.

The fresh shoots of Huazhu No. 5 (about 15 cm in length) were selected and disinfected with 1‰ KMnO_4_ solution for 30 min, followed by cutting in a black plastic cup and culture in an incubator at diurnal temperature of 26°C/22°C and under photoperiods of 16 h/8 h. When the hydroponic roots grew to about 7 cm in length, 2–6 cm root samples were obtained. The specific sampling positions are shown in Supplementary Figure 1. The tissues obtained were cut into pieces, placed into centrifuge tubes and stored in an ultra-low-temperature refrigerator at −70°C until RNA extraction.

### DNA and RNA Extraction

Ramie genomic DNA was extracted from fresh leaves of Huazhu No. 5 using a genomic DNA extraction kit (OMEGA bio-tec, CA, United States). The genomic DNA obtained was used for PCR amplification. From the tissues mentioned above, total RNA was extracted using RNAprep Pure Plant Kit (Polysaccharides and Polyphenolics-rich) (Tiangen Biotech, Beijing, China). Then use it to obtain cDNA by reverse transcription using the GoScript Reverse Transcription System (Promega, Madison, MI, United States).

### Quantitative Real-Time PCR (qRT-PCR) Analysis

The 120 candidate genes obtained from previous studies (Huang et al., 2014) were screened by semi-quantitative PCR based on their expression in five tissues of ramie, namely, bark, vein, leaf, middle stem, and root. only those expressed in the bark and vein were selected for subsequent experimental verification. Seven candidate genes were selected, gene IDs: comp37997_c0 (*BnPSP-1*), comp34113_c0 (*BnPSP-2*), comp23939_c1 (*BnPSP-3*), comp34130_c0 (*BnPSP-4*), comp23939_c3 (*BnPSP-5*), comp23939_c2 (*BnPSP-6*), and comp38891_c0 (*BnPSP-7*).

The qRT-PCR was performed on a Bio-Rad iQ5 Real-Time PCR System (Bio-Rad, CA, United States), with GAPDH gene as a reference gene (Huang et al., 2014). The relative expression levels of genes were calculated based on the methods in previous studies (Livak and Schmittgen, 2001). Statistical analysis was performed using one-way analysis of variance (SPSS, Chicago, Illinois, United States).

### Isolation and Predictive Analysis of Promoter Regions

Probable promoter region fragments were cloned by the UFW method (Myrick and Gelbart, 2002). From the start to the end of UFW cloning, the prepared system was temporarily stored at 4°C and then added into A for reactions according to the detailed operation processes (Supplementary Table 1). The primers for UFW were designed according to those in the method mentioned above, and the primer sequences are shown in Supplementary Table 2. The remaining primers (Supplementary Tables 3, 4) were designed according to the following methods. The promoter sequences were analyzed in detail online by PlantCARE^1^ for predicting their *cis*-acting elements (Magali Lescot et al., 2002).

### Construction of Promoters- and Truncated Fragments-GUS Reporter Constructs

Based on the distribution of *cis*-acting elements, a series of 5′ truncated fragments were generated for the screened *BnPSP1*, *BnPSP2*, and *BnPSP4*. The primers applied are shown in Supplementary Table 3.

The target fragments were then introduced into the binary vector pBI121 (Supplementary Figure 2) to replace CaMV 35s promoter. The primers use dare provided in Supplementary Table 4. The recombinant vectors were confirmed via sequencing. Subsequently, the corresponding plasmids were transformed into *Agrobacterium tumefaciens* GV3101 for later use.

### Transformation of Arabidopsis and Detection of Transgenic Plants

Wild-type Arabidopsis was used for transformation through the Agrobacterium-mediated floral dip method (Clough and Bent, 2010). The harvested seeds were surface sterilized, and kanamycin was used as a resistance screen. Normally grown Arabidopsis seedlings were transplanted into sterile soil to harvest T1 lines.

Genomic DNA was extracted from Arabidopsis with DNA extraction kit. The positive plants were identified by PCR. Next, the positive plants were grown individually to self-fertilize to T2 homozygous lines, and then T3 were obtained through the same process. T3 generation seeds of more than three independent transgenic Arabidopsis lines (genetically unique) were selected for each target construct.

### Histochemical Assay of GUS Activity

The GUS solution [50 μmol⋅L^–1^ phosphate buffer (pH = 7), 50 μmol⋅L^–1^ EDTA⋅2Na, 0.5 μmol⋅L^–^1 K_3_[Fe(CN)_6_], 0.5 μmol⋅L^–1^ K_4_[Fe(CN)_6_,] ⋅3H_2_O, 0.1% Triton X-100, 20% CH_3_OH and 2 μmol⋅L^–1^ X-gluc] was modified and prepared based on the methods of Jefferson et al. (Jefferson et al., 1987).

Seedlings (1, 3, 5, 7, and 10 days after germination) of each transgenic line of wild-type and T3 transgenic Arabidopsis were immersed in GUS staining solution and placed in a dark incubator at 37°C for 12 h. The corresponding mature tissues or organs (leaf, flower and silique) are also stained as above. Later, the GUS staining solution was removed, and the samples were decolorized with 75% ethanol in an incubator at 37°C for 12 h, during which the 75% ethanol was replaced two to three times. The whole images were generated by stereo fluorescence microscopy (SZX16, Olympus, Japan), the specific expression sites were observed, and their images were collected.

### Measurement of GUS Activity

Target T3 Arabidopsis seeds were surface sterilized and grown on 1/2 MS medium supplemented with kanamycin (40 ng/μL) for hormone and simulated drought treatment experiments. After 12 days, the seedlings with consistent growth were transferred intoculture dishes with filter paper and treated with dH_2_O (control), 10 μmol⋅L^–1^ GA3, 10 μmol⋅L^–1^ indoleacetic acid (IAA), 100 μmol⋅L^–1^ abscisic acid (ABA), 20% PEG6000, 200 μmol⋅L^–1^ CuSO_4_ and 100 μmol⋅L^–1^ 6-BA for 6 h at 22°C under white light. After the surface liquid has dried, approximately 100 mg of the treated sample was collected into a centrifuge tube and frozen in liquid nitrogen.

GUS protein was extracted from all treated samples using GUS protein extraction buffer (50 mM NaH_2_PO_4_, pH 7.0, 10 mM EDTA,0.1% sodium lauryl sarcosine, pH 8.0, 10 mM β-mercaptoethanol, 0.1% Triton X-100). After centrifugation at 12,000 × g and 4°C for 10 min, the supernatant was collected, and protein concentration of the extract was determined by the method of Bradford (1976). The enzyme reaction assay and fluorogenic reaction were performed as reported (Jefferson et al., 1987). The fluorescence intensity of the reaction solution was measured using a multifunctional microplate reader (EnSpire, PerkinElmer, United States) with different wavelengths of 365 nm (excitation) and 455 nm (emission). The concentration of 4-methylumbelliferyl glucuronide was calculated according to a standard curve. Finally, the GUS activity in transgenic plants was obtained (Jefferson et al., 1987). SPSS 20.0 was employed for data analysis, and Graph Pad Prism 5.0 was used for charting.

## Results

### Screening of Genes Preferentially Expressed in Ramie Bark

According to the results of semi-quantitative PCR screening, seven genes meet the requirements that preferentially expressed in the bark and vein. The corresponding genes were named as *BnPSP-1*, *BnPSP-2*, *BnPSP-3*, *BnPSP-4*, *BnPSP-5*, *BnPSP-6*, and *BnPSP-7* and used for the follow-up verification experiments. In order to identify these seven genes, we aligned these seven sequences (*BnPSP-1*, *BnPSP-2*, *BnPSP-3*, *BnPSP-4*, *BnPSP-5*, *BnPSP-6*, and *BnPSP-7*) with the NR database using blastx. It was found that *BnPSP-1* and *BnPSP-7* were two-component response regulator (*ARR*) genes in the cytokinin signaling pathways. The alignment results showed that the *BnPSP-1* (*B-ARR*) and *BnPSP-7* shared 85.16 and 84.23% sequence similarity with mulberry homologous genes, respectively. *BnPSP-4* was identified as an IAA gene and its sequence was 81.00% identical to the sequence of indole-3-acetic acid-amide synthetase gene of mulberry. *BnPSP-2*, *BnPSP-3*, *BnPSP-5*, and *BnPSP-6* are *AUX/LAX* auxin transport genes.

**FIGURE 1:**
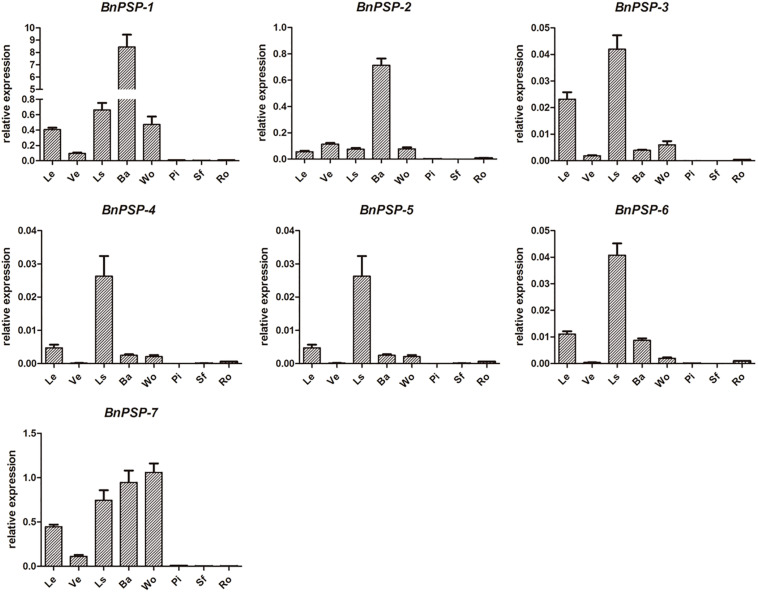
QRT-PCR results of seven ramie endogenous genes in different organs or tissues. BnPSP-1, BnPSP-2, BnPSP-3, BnPSP-4, BnPSP-5, BnPSP-6, and BnPSP-7, in eight different organs or tissues, respectively. Data were presented as the mean ± SE of three separate measurements for each independent line. Le, Leaf; Ve, Vein; Ls, Petiole; Ba, Bark; Wo, Wood; Pi, Pith; Sf, Cotton pulp; Ro, Root.

QRT-PCR was performed for parts of ramie to explore the expression patterns of selected genes. As shown in Figure 1, the relative expression levels of *BnPSP-1*-*BnPSP-7* genes were different. And the relative expression levels of the same gene in different tissues or organs are also different. Specifically, the relative expression level of *BnPSP-1* was the highest in the external stem, 14 times greater than the second-highest tissue of petiole. The relative expression level of *BnPSP*-2 was also highest in the external stem, 6 times that of the next highest, vein. The relative expression level of *BnPSP*-4 was 43.40 in the xylem, 3 times that of the level in the bark. These results suggest that *BnPSP-1* and *BnPSP-2* are preferentially expressed in the bark, and *BnPSP*-4 has the highest relative expression level and is preferentially expressed in the wood. The relative expression levels of *BnPSP-3*, *BnPSP-5*, and *BnPSP-6* are low, and *BnPSP-7* does not have preferential expression. Finally, *BnPSP-1*, *BnPSP-2*, and *BnPSP-4* were confirmed as the downstream target genes of the cloned promoters for the follow-up experiments.

### Cloning and Sequence Analysis of Candidate Promoters

**FIGURE 2:**
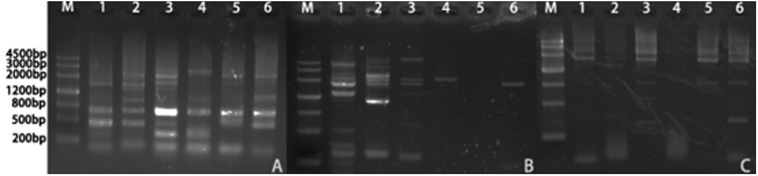
Products of Ramie promoters. **(A)** PCR product of P_*PSP1*_; **(B)** PCR product of P_*PSP2*_; **(C)** PCR product of P_*PSP3*_; M: 4,500 bp Ladder; 1: Annealing temperature 55°C; 2: Annealing temperature 56°C; 3: Annealing temperature 57°C; 4: Annealing temperature 58°C; 5: Annealing temperature 59°C; 6: Annealing temperature 60°C.

We isolated the promoter regions of *BnPSP-1*, *BnPSP-2*, and *BnPSP-4* using the UFW method. According to the cloning results (Figure 2), TA cloning was performed on the PCR products with multiple bands at appropriate temperatures, and the initial promoter sequence was obtained after sequencing. Next, this sequence was applied to design the *cis*-specific primer, and the genomic DNA sequence-specific primer served as the *trans*-primer. After verification by sequencing, 1,621, 2,299, and 2,254 bp sequences upstream of corresponding start codons were obtained successfully, which contained the promoters of *BnPSP-1*, *BnPSP-2*, and *BnPSP-4* and named as *P*_*PSP1*_, *P*_*PSP2*_, and *P*_*PSP4*_, respectively (GenBank accession numbers: MT136746; MT136747; MT136748).

The promoter length of the known sequences was predicted online^2^. The results (Supplementary Figure 3) predicted that the transcription start site (TSS) of *P*_*PSP1*_ was located −491 bp upstream of the start codon, P_*PSP2*_ at −1,755 bp upstream, and P_*PSP4*_ at −259 bp upstream, consistent with 5′-untranslated regions in the upstream of the start codons. Therefore, the targeted analysis was performed for such sites in subsequent verification tests. The Genome Information Database System from the Institute of Crop Science of the Japanese National Agriculture and Food Research Organization^3^ was used to discover putative *cis*-acting elements.

The *cis*-acting elements in *P*_*PSP1*_ included hormone-responsive elements, Cu^2+^ responsive elements, dehydration-induced and darkness-induced responsive elements and S responsive elements. Deletion analysis was used to verify the function of predicted elements. The detailed name, sequence information and site of the elements are shown in Supplementary Table 5. For *P*_*PSP2*_, there were *cis*-acting elements such as hormone-responsive elements, dehydration-induced and darkness-induced responsive elements, drought-responsive elements, photo-responsive elements, ion responsive elements and sucrose responsive elements. The detailed information is shown in Supplementary Table 6. The *cis*-acting elements on *P*_*PSP4*_ included photo-responsive elements, ion responsive elements, dehydration-induced and darkness-induced responsive elements and hormone-responsive elements (Supplementary Table 7).

### GUS Expression Patterns in Transgenic Arabidopsis

**FIGURE 3:**
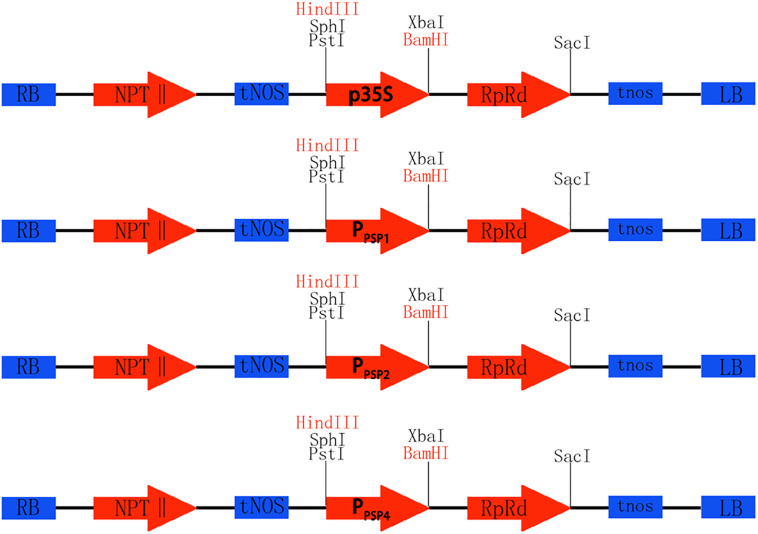
Construction of promoter vector. Rb and Lb are Terminal repeats, NPTII is Kanamycin resistance gene, tNOS is a Nopaline synthase terminator, GUS is Reporter gene, Restriction sites of HindIII and BamHI, p35S is the original promoter, P_PSP1_, P_PSP2_, and P_PSP4_ are endogenous promoters of ramie.

**FIGURE 4:**
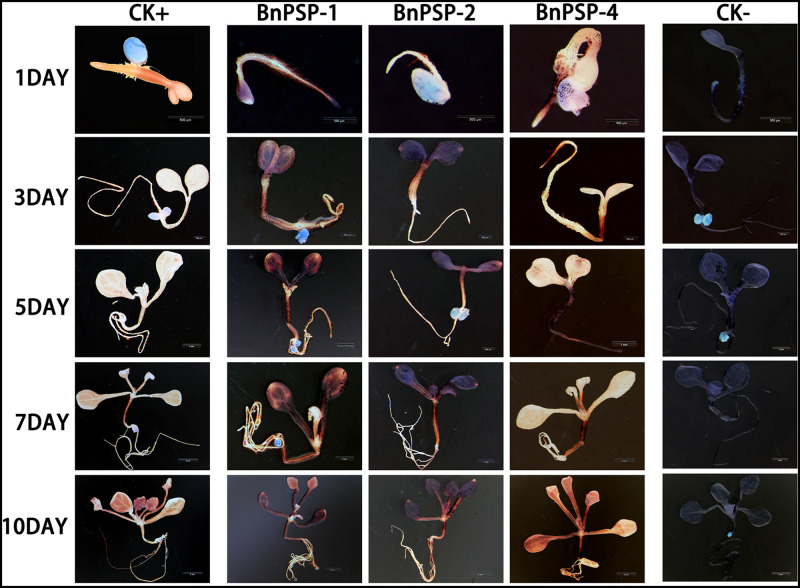
Representative GUS histochemical assay staining in different development stages (1-, 3-, 5-, 7-, and 10-day after germination) of Arabidopsis seedlings. CK+ indicated transgenic Arabidopsis seedlings carrying CaMV35S promoter construct; CK– indicated wild-type. P_PSP1_, P_PSP2_, and P_PSP4_ represent Arabidopsis seedlings of three independent promoter vectors.

To investigate the expression pattern of the cloned promoters, the promoter sequences were introduced into pBI121 to construct expression vectors pBI121-*P*_*PSP1*_, pBI121-*P*_*PSP2*_, and pBI121-*P*_*PSP4*_ (Figure 3). Then these vectors were transformed into *Agrobacterium* GV3101 by electroporation and confirmed by sequencing. We used *Agrobacterium tumefaciens*-floral dip method to obtain transgenic lines carrying *P*_*PSP1*_, *P*_*PSP2*_, and *P*_*PSP4*_ (CaMV35 promoter as the positive control, wild-type as the negative control). The 1, 3, 5, 7, and 10-day-old Arabidopsis seedlings were stained and observed (Figure 4). *P*_*PSP1*_-driven GUS reporter gene was initially expressed at every site of germinated Arabidopsis seeds but was only expressed in the vein, stem and root of 3–10 days seedlings. *P*_*PSP2*_-driven GUS reporter gene was also initially expressed at every site of germinated seeds but was expressed in the cotyledon growth point and root of the seedlings at 3–10 days of development.

**FIGURE 5:**
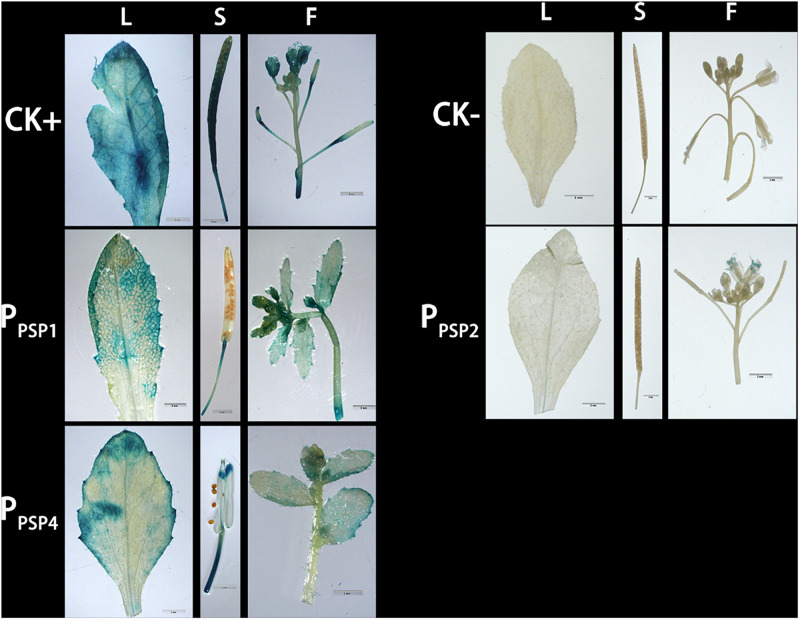
Representative GUS histochemical assay staining in different mature organs of Arabidopsis. CK+ indicated transgenic Arabidopsis seedlings carrying CaMV35S promoter construct; CK– indicated wild-type; P_PSP1_, P_PSP2_, and P_PSP4_ represent Arabidopsis lines of three independent promoter vectors. L, Leaf; S, Silique; F, Flower.

GUS expression was also observed in the stem and petiole of 5–10-day-old seedlings. *P*_*PSP4*_-driven GUS reporter gene was expressed in all tissues of 10 days seedlings. To further understand the expression of each promoter in mature plants, transgenic plants were transplanted into soil. The leaves, siliques and flowers of mature Arabidopsis were stained and observed. According to the staining results (Figure 5), *P*_*PSP1*_-driven GUS was only expressed in the vein and stem, P_*PSP2*_-driven GUS expression was observed only in the floral organ, and *P*_*PSP4*_-driven GUS expression was visible at the leaf, stem, silique and flower. We integrated the staining results of both seedlings and mature plants and confirmed that *P*_*PSP1*_-driven GUS reporter gene was specifically expressed in the phloem. Therefore, an in-depth study was focused on this promoter.

### Construction of Truncated Vectors of Promoters and Functional Verification

**FIGURE 6:**
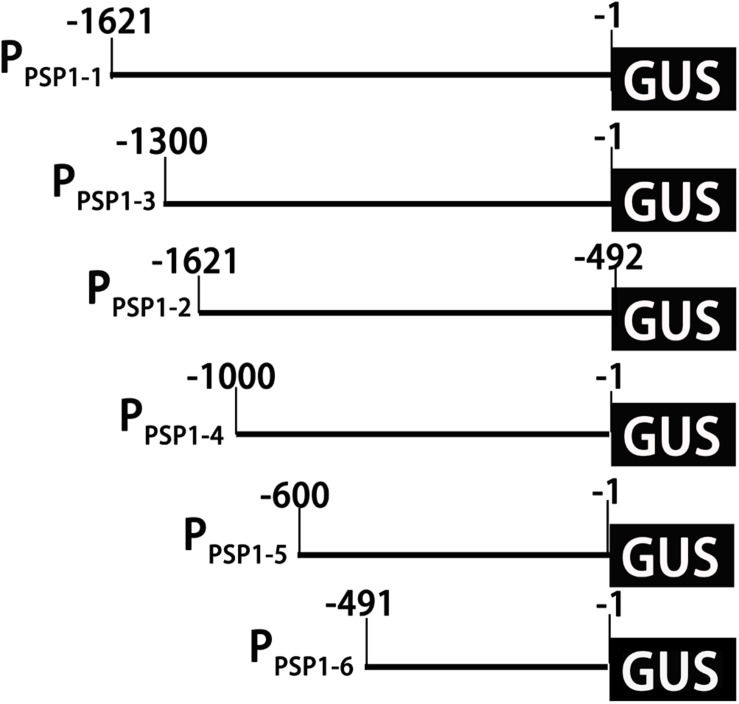
Schematic diagram of truncated fragments in the P_PSP1_ region. The initiation codon was defined as +1. The number indicated the length of the 5′-truncated fragments.

**FIGURE 7:**
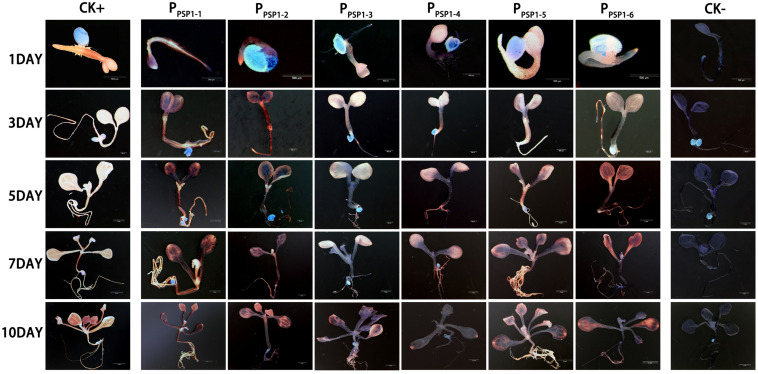
Representative GUS histochemical assay staining in different development stages (1-, 3-, 5-, 7-, and 10-day after germination) of *Arabidopsis* seedlings. CK+ indicated transgenic *Arabidopsis* seedlings carrying *CaMV35S* promoter construct; CK***–*** indicated wild-type. *P_*PSP*1–1_*, *P_*PSP*1–2_*, *P_*PSP*1–3_*, *P_*PSP*1–4_*, *P_*PSP*1–5_*, and *P_*PSP*1–6_* represents P_*PSP1*_ and its truncated promoter transgenic *Arabidopsis* seedlings.

According to the location of predicted *cis*-acting elements, the *P*_*PSP1*_ was properly truncated into five fragments and introduced into pBI121 to construct fusion vectors with GUS, respectively. The construction results are provided in Figure 6, full-length pBI121-*P*_*PSP1*_ was named *P_*PSP*1–1_*, and the other vectors were named *P_*PSP*1–2_*, *P_*PSP*1–3_*, *P_*PSP*1–4_*, *P_*PSP*1–5_*, and *P_*PSP*1–6_*, respectively. The bioinformatics analysis of *P*_*PSP1*_ predicted that the TSS was located at −491 bp upstream of the initiation codon, so we constructed the deletion vector *P_*PSP*1–2_* with the −1,621 to −492 bp region. The above vectors were transformed into Arabidopsis, for which the staining results in different development stages are shown in Figure 7. As for *P_*PSP*1–2_*, GUS staining was visible in the outer edge of the leaves, stems and roots of 1–5-day-old seedlings. All the new leaves of the 10-day-old seedlings were stained, while other stained areas were the same as those of *P_*PSP*1–1_*. In terms of *P_*PSP*1–3_*, all the leaves and roots, instead of the stems, of 1–7-day-old seedlings were stained. In 10-day-old seedlings, all the new leaves and the outer edge of other leaves were stained, while the stems lost expression. For *P_*PSP*1–4_*, all the leaves and roots, except the stems, of 1–7-day-old seedlings were stained. In 10-day-old seedlings, only the leaf growth points were stained. As for *P_*PSP*1–5_*, every tissue of 1–5-day-old seedlings was stained. In 7-day-old seedlings, the staining results for the leaf organs were changed; that is, only the growth points and veins were stained, and the remaining stained areas remained unchanged. In 10-day-old seedlings, the staining results were the same as those of *P_*PSP*1–1_* and *P_*PSP*1–2_*. For *P_*PSP*1–6_*, all the sites except for the stem of 1–7-day-old seedlings were stained, while only the roots, leaves and growth points of new leaves of 10-day-old seedlings were stained.

**FIGURE 8:**
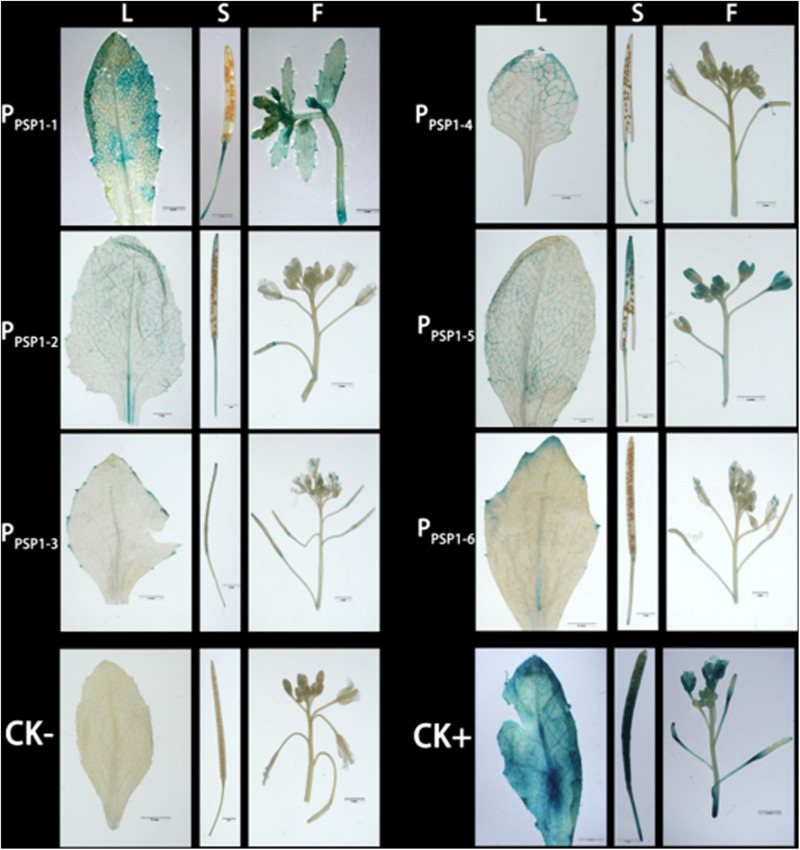
Representative GUS histochemical assay staining in different mature organs of Arabidopsis. CK+ indicated transgenic Arabidopsis seedlings carrying CaMV35S promoter construct; CKv indicated wild-type; P_*PSP*1–1_, P_*PSP*1–2_, P_*PSP*1–3_, P_*PSP*1–4_, P_*PSP*1–5_, and P_*PSP*1–6_ represents P_*PSP1*_ and its truncated promoter transgenic Arabidopsis lines. L, Leaf; S, Silique; F, Flower.

Staining analysis was also performed in mature organs of Arabidopsis (Figure 8). In the lines carrying *P_*PSP*1–2_* construct, GUS expression was detected in the veins, silique tips, and floral organs. In the lines carrying *P_*PSP*1–3_* construct, weak GUS expression was detected in the outer edge of leaves, silique tips and floral organs. In the lines carrying *P_*PSP*1–5_* construct, GUS activity was detected in the veins, siliques and floral organs. In the lines carrying the *P_*PSP*1–6_* construct, GUS expression activity was detected in the outer edge of leaves and floral organs without the siliques. The GUS staining results of the above promoters, except for *P_*PSP*1–4_*, in the transgenic *Arabidopsis* lines were consistent with the growth and development trends of the stained areas of the seedlings.

### Quantification of GUS Activity in Transgenic Arabidopsis Lines

Randomly selected transgenic Arabidopsis lines carrying *P*_*PSP1*_ and each truncated T3 (three independent lines for each candidate gene) were used to assess the impacts of hormone and simulated drought treatment on the activity of the *P*_*PSP1*_ series of sequences. At the same time, the GUS activity of the samples was measured under different exogenous hormones and drought simulation conditions.

**FIGURE 9:**
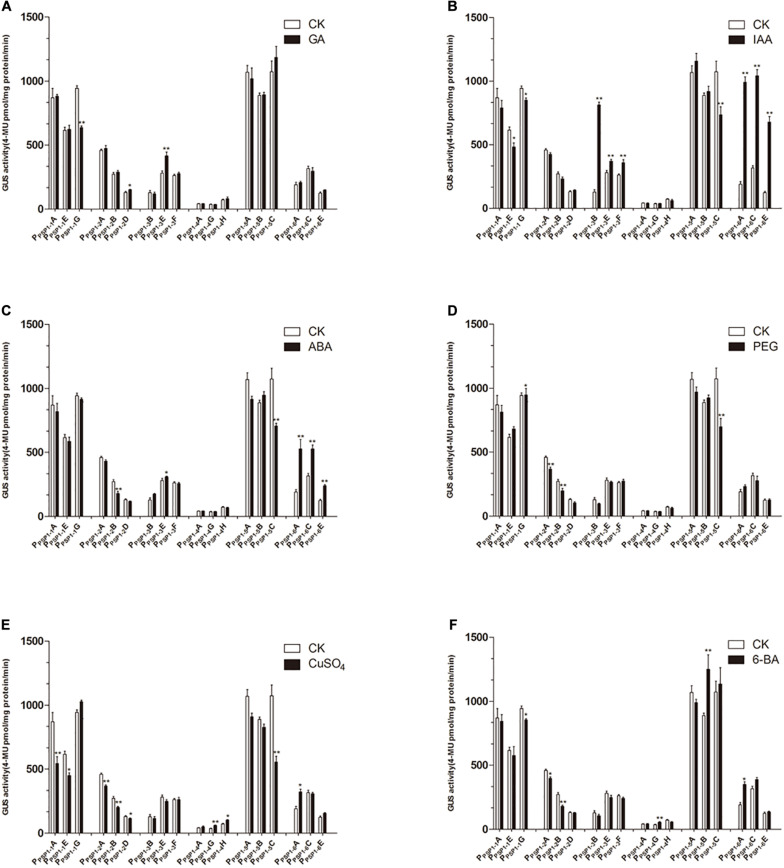
GUS activities of transgenic Arabidopsis seedlings carrying different constructs under different treatments. CK means treated with dH_2_O. **(A–F)** Denoted GUS activity of Arabidopsis seedlings carrying P_*PSP*1–1_, P_*PSP*1–2_, P_*PSP*1–3_, P_*PSP*1–4_, P_*PSP*1–5_, and P_*PSP*1–6_ constructs under GA, IAA, ABA, PEG, CuSO_4_, and 6-BA treatment, respectively. Each construct corresponds to three independent transgenic lines. The data were presented as the mean ± SE. *p < 0.05 and **p < 0.01.

Compared to the control, the GA3 treatment affected the GUS activity of the transgenic lines to a lesser extent (Figure 9A). The IAA treatment of *P_*PSP*1–3_* and *P_*PSP*1–6_* significantly increased the GUS expression level of each line (Figure 9B). *P_*PSP*1–6_* was also significantly induced by ABA (Figure 9C). PEG-simulated drought stress (Figure 9D) had no significant effect compared to the control on lines carrying the *P_*PSP*1–1_*, *P_*PSP*1–3_*, *P_*PSP*1–4_*, and *P_*PSP*1–6_* constructs. Treatments with CuSO_4_ and 6-BA resulted in no change or inconsistent changes in GUS expression levels across all lines (Figures 9E,F). One exception was the copper significantly decreased expression of all three lines of *P_*PSP*1–2_*.

## Discussion

### Cloning and Application of Phloem-Specific Promoters

Gene expression in higher plants is mainly regulated at the transcription level, a process affected by the coordination between multiple *cis*-acting elements in the promoter regions and distal *trans*-acting factors (Kuhlemeier et al., 1987). Constitutive promoters have many disadvantages for the genetic improvement of crops. For example, they can cause extra metabolic burden to the plants and induce gene silencing (Shelton et al., 2001; Hsu et al., 2008). Therefore, tissue specificity promoter has received more attention (Corrado and Karali, 2009; Golan et al., 2013). During the discovery of new phloem-specific promoters, more scholars focus on the functional analysis of promoters and their application in resistance to diseases and pests as well as plant nutrition. For instance, the promoters applied for crop insect-resistant genetic engineering include the enhanced pumpkin *PP2* promoter (Guo et al., 2004), the rice sucrose synthase gene *RSs1* promoter driving the expression of insecticidal proteins (Saha et al., 2007), and phloem-specific promoters applied for resistance against bollworm in cotton (Shah et al., 2011). Phloem-specific promoters from Arabidopsis may be useful for engineering phytoplasma-resistant transgenic strawberries (Zhao et al., 2004). Dutt et al. (2012) confirmed phloem-specific expression in Mexican lime from four transgenic promoters. Zou et al. (2017) selected phloem-specific promoter GRP1.8 to drive the expression of cecropin B, which lowered susceptibility to Huanglongbing.

Studies on the application of phloem-specific promoters are dominated by those on pest and disease resistance, with promoters mostly verified in model crops. However, there are studies on tissue-specific promoters in ramie. Ma cloned the ramie mannanase *BnMAN1* gene promoter and presumed based on the types of response elements in the promoter that it may participate in hormonal regulation (Ma et al., 2018). Guo cloned 15 ramie phloem protein 2 (*BnPP2-BnPP215*) genes (Guo et al., 2018) and conducted promoter functional analysis in the model organism Arabidopsis (Guo et al., 2019a). Developing phloem-specific promoters has practical significance in the research and application in fiber development and pest and disease resistance of ramie.

Obtaining accurate sequence via cloning is a critical step in studies on either specific promoters or corresponding gene functions. In this experiment, we applied a laboratory-modified UFW method to clone promoter fragments (Chen et al., 2015; Guo et al., 2018), and three promoters (*P*_*PSP1*_, *P*_*PSP2*_, and *P*_*PSP4*_) were obtained successfully. Although the upstream promoter sequences of the target genes of plant materials without genomic data can be successfully obtained using this method, it has limitations of being time-consuming and having complicated experimental procedures. The experimental procedures of promoter cloning may be simplified by FPNI-PCR in the future (Wang et al., 2011). Following the publication of genomic data of ramie (Liu et al., 2017; Luan et al., 2018), promoter sequences of ramie can be obtained directly by designing PCR primers based on desired genome sequences. However, our approach still has reference value for many species in which valid genomic data are difficult to obtain.

### GUS Expression Pattern in PPSP1 and Its Truncated Promoter

There is a two-component regulatory system constituted by two kinds of transcription regulators [namely A-type ARR (inhibit transcriptional activity) and B-type ARR (enhance transcriptional activity)] in the cytokinin signaling pathway of higher plants (Hwang and Sheen, 2001), which were first reported in Arabidopsis (Lohrmann et al., 2001; Sakai and Aoyama, 2010). With the development of genome sequencing technology, C-type ARR (negative regulator) has been identified, which has similar functions to A-type ARR and can inhibit signal transduction (Gattolin et al., 2005). Sequence alignments reveal that *BnPSP-1* is the most homologous to *ARR12* in Arabidopsis, so it belongs to B-type ARR. In this study, real-time fluorescence quantitative analysis and promoter cloning analysis were performed for *BnPSP-1* [cytokinin response regulator of ramie (*BnARR*)], and the results showed that the gene could be expressed in every tissue site (Figure 1), consistent with the findings of ARR in Arabidopsis (Argyros et al., 2008), *Populus* (Ramírez-Carvajal et al., 2009), and *Oryza sativa* (Cheng et al., 2010). Promoter analysis by GUS reporter staining results for transgenic Arabidopsis indicated that GUS activity can be detected in the root, cotyledon and vein of mature leaf of seedlings as well as the floral organ of the lines carrying *P_*PSP*1–1_*, *P_*PSP*1–2_*, and *P_*PSP*1–5_* constructs (Figures 7, 8), which are in line with the studies on Arabidopsis (Yoshinori et al., 2004) and lotus rhizome (Prasad Lohar et al., 2004). We discovered the key elements related to phloem-specific expression in the above promoter regions: (EBOXBNNAPA) (Hartmann et al., 2005), (−300ELEMENT) (Colot et al., 1987), and (OSE1ROOTNODULE) (Vieweg et al., 2004).

GUS was expressed in the root, leaf margin and floral organ in transgenic Arabidopsis carrying *P_*PSP*1–3_* construct, and its expression activity was repressed compared with that in other truncated vectors, suggesting that suppression elements may exist in the −1,300 to −1,000 bp region of the promoter sequence. However, no known repressor and other functional elements were predicted in the region (Meagher et al., 2009). Our data suggest that there are unidentified suppression elements in this region, and in-depth studies are needed to determine the specific information of the elements. GUS expression was only detected in the root tip and leaf growth point of Arabidopsis seedlings transformed with the *P_*PSP*1–4_* construct. When the plants were mature, the GUS expression could be detected in the vein, silique and floral organ. The presumed reason may be that the promoter-driven gene expression has spatiotemporal characters. Promoters only drive the GUS expression at the growth points of transgenic Arabidopsis seedlings, and its ability to drive the GUS expression is enhanced as the plant is growing. It also conforms to the character that some promoters have tissue specificity and spatiotemporal specificity at the same time (Zhao et al., 1994). In addition, we could determine the specific site and stage of GUS expression by sampling and observation of Arabidopsis during the whole growth period, and observe whether the expression of the promoters is spatiotemporal or inducible by some enzymes through the treatment of the promoters with relevant enzymes synthesized in certain stages.

When the promoters were truncated to *P_*PSP*1–6_*, GUS was expressed only in the root, cotyledon and mature leaf, illustrating that there is tissue-specific promoter-related elements in the −600 to −491 bp region of the promoter sequence. According to the GUS expression results of *P_*PSP*1–2_* and *P_*PSP*1–5_* promoters, we could construct truncated vectors or point mutant vectors in the −600 to −492 bp region of *P*_*PSP1*_ sequence and transform them into Arabidopsis to detect the GUS expression, thus determining whether this sequence fragment is the key region maintaining the tissue specificity of the promoters and providing optimized results for subsequent studies. A 5′-untranslated region was predicted in the *BnPSP-1* gene (Supplementary Figure 2). In this study, the *P*_*PSP1*_ fragment was truncated to the 491 bp region to obtain *P_*PSP*1–6_* according to the distribution of predicted elements, which could still drive the GUS expression in Arabidopsis. The possible reason is that the core promoter element (TATA-BOX) exists in the −491 to −1 bp region of the sequence.

### The Activity of PPSP1 and Its Truncated Promoter Under Different Treatments

*P*_*PSP1*_ promoter function was further verified using a quantitative analysis of GUS expression in transgenic lines under hormone and simulated drought treatment. The exogenous application of cytokinin (6-BA), a type of plant regulator, can increase *AtARR1* and *AtARR10* gene expression (Kristine et al., 2013). In this study, however, the *Arabidopsis* lines carrying *P*_*PSP1*_ or truncated fragments exhibited little response to 6-BA (Figure 9F). There are two likely reasons: the variance between species or promoter specificity is not completely consistent with the function of downstream genes, which was also reported in grapevine (Yu et al., 2017).

Considering the presence of the auxin response factor (ARF) (Yoshiaki et al., 2005), auxin (IAA) was applied to the transgenic lines in this study. Auxin had effects on the activity of *P_*PSP*1–1_*, *P_*PSP*1–3_*, and *P_*PSP*1–6_* promoters, while other promoters were insensitive (Figure 9B). *P_*PSP*1–2_* promoter is insensitive to auxin likely because it lacks the predicted auxin response element that is located at the −304 bp region. However, auxin had no effects on lines carrying *P_*PSP*1–4_* and *P_*PSP*1–5_* constructs. The reason may be that there is an interaction between auxin and cytokinin (Jirí et al., 2004; Cheng et al., 2013), with elements responsive to auxin inhibition in the −1,300 to −600 bp region.

Only the *P_*PSP*1–6_* promoter had a strong response to ABA treatment (Figure 9C), potentially because of the ABA response element (MYB1AT) (Hobo et al., 2010) in the −491 to −1 bp region. It is presumed that the repressor in the −1,621 to −492 bp region inhibits the response to ABA. The CuSO_4_ treatment was performed given the predicted copper response element (CURECORECR) (Janette et al., 2005). Copper induced the activity of *P_*PSP*1–2_* while the remaining promoters were insensitive (Figure 9E). It was inferred from the results that the copper response element in the −491 to −1 bp region may activate the promoter, and activity declines if the fragment is lost. However, the specific role of the element needs to be identified in follow-up experiments. The functional analysis of *P*_*PSP1*_ and its truncated fragments displayed that *P_*PSP*1–1_* and *P_*PSP*1–5_* did not have strong responses to various treatments but had the characters of tissue-specific expression, so these two promoters can be explored in follow-up.

## Conclusion

In this study, we applied the modified UFW method to clone three candidate endogenous promoters (*P*_*PSP1*_, *P*_*PSP2*_, and *P*_*PSP4*_) preferentially expressed in ramie bark and transformed them into Arabidopsis using *Agrobacterium* to verify the expression pattern. One phloem-specific promoter, *P*_*PSP1*_, was identified. Under various treatments, Arabidopsis transformed with *P_*PSP*1–2_* (−1,621 to −429 bp) and *P_*PSP*1–5_* (−600 to −1 bp) truncated promoters had stable phloem specificity. The isolation and identification of *P_*PSP*1–5_-BnPSP-1* not only provides candidate promoters for the development of ramie molecular biology and targeted improvement of fiber quality but also enriches the plant promoter database.

## Data Availability Statement

The raw data supporting the conclusions of this article will be made available by the authors, without undue reservation.

## Author Contributions

YW and BW conceived and designed the experiments. YW, YB, YZ, PG, DP, and BW performed the experiments and analyzed the data. YW, YB, and BW wrote the manuscript. All authors read and approved the manuscript.

## Conflict of Interest

The authors declare that the research was conducted in the absence of any commercial or financial relationships that could be construed as a potential conflict of interest.

## Footnotes

1 http://bioinformatics.psb.ugent.be/webtools/plantcare/html/

2 http://linux1.softberry.com/

3 https://sogo.dna.affrc.go.jp/cgi-bin/sogo.cgi?lang=en

## Supplementary Material

Supplementary Figure 1 Schematic diagram of tissues for RNA extraction. **(A–H)** represented petiole, main vein, leaf (without main vein), phloem, xylem, pith, root, respectively.

Supplementary Figure 2 The T-DNA region of pBI121 binary plasmid.

Supplementary Figure 3 Prediction of position and length of PPSP1, PPSP2, and PPSP4 promoters. **(A)** PPSP1; **(B)** PPSP2; **(C)** PPSP4.

Supplementary Table 1 The PCR procedure of UFW.

Supplementary Table 2 The primers information of UFW to isolate three promoter regions.

Supplementary Table 3 The primers information of promoter regions detection.

Supplementary Table 4 The primers for constructing binary expression vectors.

Supplementary Table 5 Putative cis-acting elements of PPSP1.

Supplementary Table 6 Putative cis-acting elements of PPSP2.

Supplementary Table 7 Putative cis-acting elements of PPSP4.
