# Supplementary figures and images for: Promoter PPSP1–5-BnPSP-1 From Ramie (Boehmeria nivea L. Gaud.) Can Drive Phloem-Specific GUS Expression in Arabidopsis thaliana
Source: Front Genet. 2020 Dec 16;11:553265. doi: 10.3389/fgene.2020.553265 (PMC7772962; doi:10.3389/fgene.2020.553265)

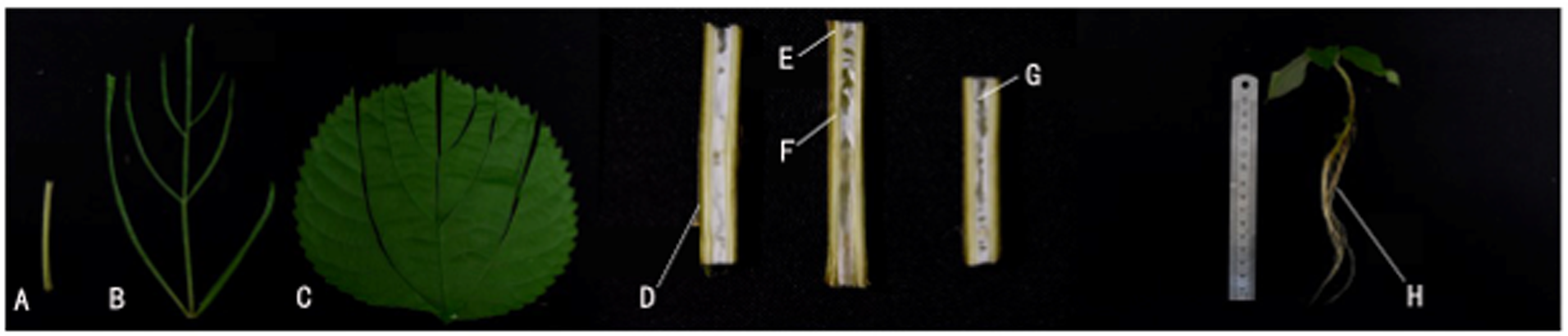

Supplement: Supplementary Figure 1 — Schematic diagram of tissues for RNA extraction. (A–H) represented petiole, main vein, leaf (without main vein), phloem, xylem, pith, root, respectively. [file Image_1.TIF]

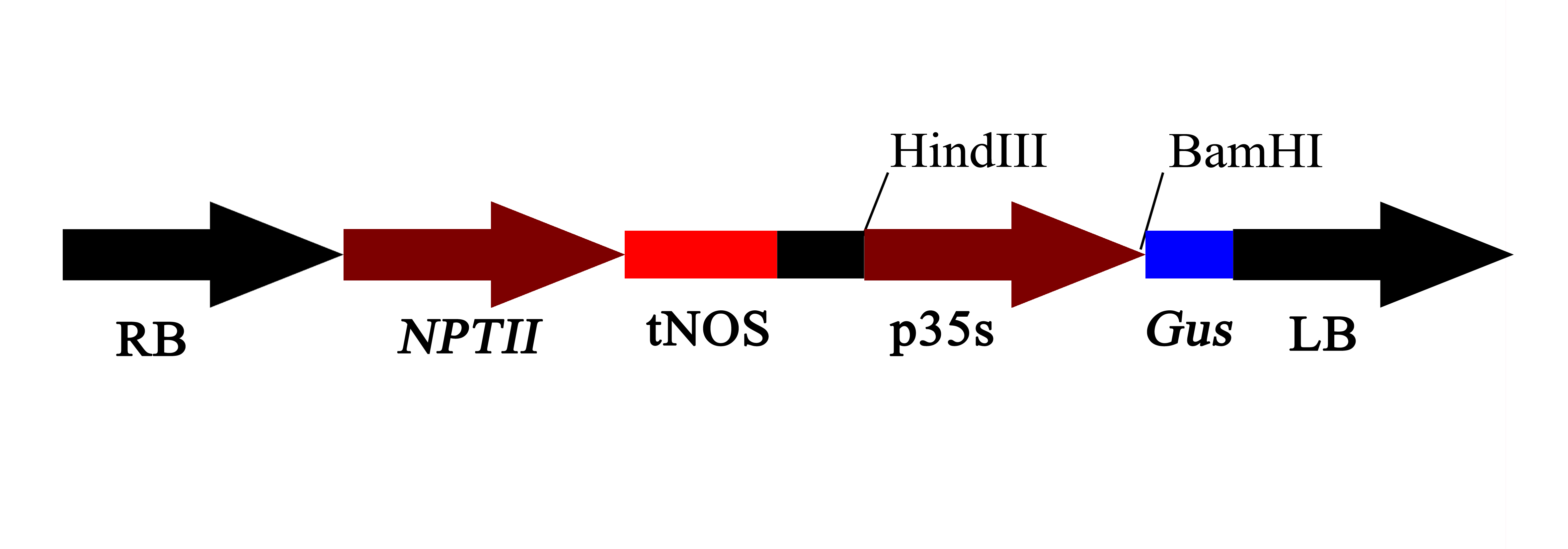

Supplement: Supplementary Figure 2 — The T-DNA region of pBI121 binary plasmid. [file Image_2.TIF]

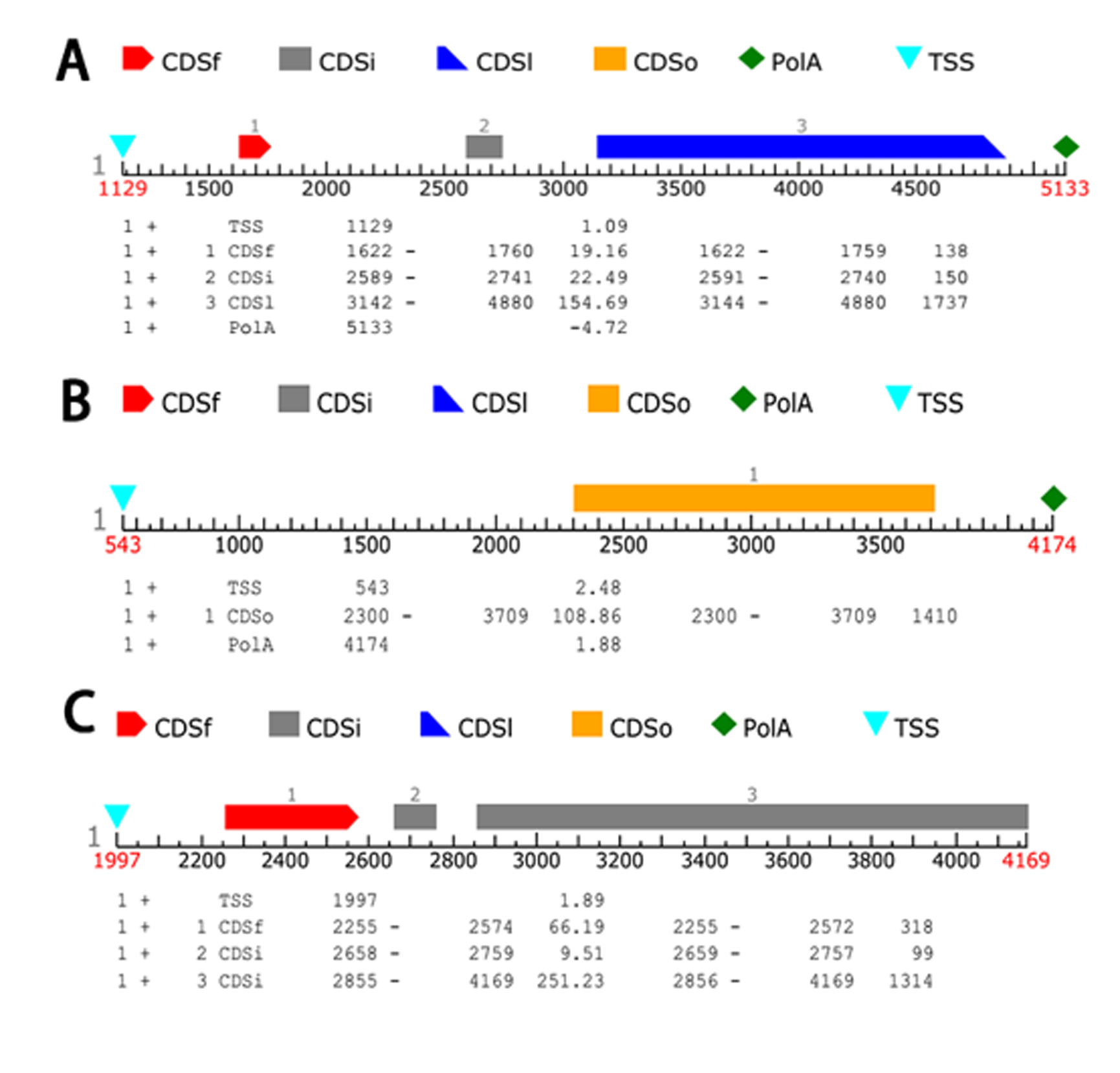

Supplement: Supplementary Figure 3 — Prediction of position and length of PPSP1, PPSP2, and PPSP4 promoters. (A) PPSP1; (B) PPSP2; (C) PPSP4. [file Image_3.TIF]
