# Supplementary material for: Promoter PPSP1–5-BnPSP-1 From Ramie (Boehmeria nivea L. Gaud.) Can Drive Phloem-Specific GUS Expression in Arabidopsis thaliana
Source: Front Genet. 2020 Dec 16;11:553265. doi: 10.3389/fgene.2020.553265 (PMC7772962; doi:10.3389/fgene.2020.553265)
Supplement: Supplementary Table 1 — The PCR procedure of UFW. [file Table_1.docx]

**Supplementary Data**

This section contains data that may be included in the online version of the journal and is cited in the main text body.

Supplementary Table 1. The PCR procedure of UFW

| A Starting mix | | B Exo I digestion-1 | | C Tagged-random priming | | D Exo I digestion-2 | | E Standard PCR | |
| --- | --- | --- | --- | --- | --- | --- | --- | --- | --- |
| Items | Volume (μl) | Items | Volume (μl) | Items | Volume (μl) | Items | Volume (μl) | Items | Volume (μl) |
| (50-100ng)DNA | 1.0 | Exo I | 1.0 | Primer2(10μM) | 1.5 | Exo I | 2.0 | Primer3(10μM) | 1.0 |
| 10×PCRBuffer | 3.0 | 10×PCRBuffer | 0.5 | 10×PCRBuffer | 0.5 | 10×PCRBuffer | 0.5 | 10×PCRBuffer | 0.5 |
| 10Mm dNTP Mix | 4.8 | ddH_2_O | 3.0 | 10Mm dNTP Mix | 3.0 | ddH_2_O | 2.5 | Primer4(10μM) | 1.0 |
| Primer1(10μM) | 0.6 |  |  |  |  |  |  | ddH_2_O | 2.3 |
| LA-Taq(2.5 units) | 0.5 |  |  |  |  |  |  | LA-Taq(2.5 units) | 0.2 |
| ddH_2_O | 20.1 |  |  |  |  |  |  |  |  |
| Manipulation | | Manipulation | | Manipulation | | Manipulation | | Manipulation | |
| Cold-startdenature at 95 °C for 5 min60 °C for 30 s68 °C, 2 minpause at 37 °C, add B | | (1)37℃ for 31 min(2) pause at the end, add C | | Cold-startdenature at 95 °C for 5 min68℃ to 20℃declining 0.2 °C per 6 seconds for rampingpause at 37℃, add D | | (1)37℃ for 46 min(2)68℃ for 15 min(3)80℃ for15 min(4)95℃ for 3 min(5)65 °C to 45 °C, declining 0.2 °C per 3 seconds for ramping(6)68℃ for 2 min（7）pause and add E | | (1) denature at 95 °C for 3 min(2)95℃, 30 s(3)58℃, 30 s(4)72℃, 3 min(5) run 40 cycles of (2)-(4)(6)68 ℃ for 10 min | |

Supplementary Table 2. The primers information of UFW to isolate three promoter regions

| Name | Primer sequence(5´-3´) |
| --- | --- |
| *BnPSP-1*-1 | CTTTCCCACTCTCTTCGCTCTCA |
| *BnPSP-1*-2 | CCAATATAAAACTACCACNNNNNNNNNN |
| *BnPSP-1*-3 | AACTTCGCATTAACTCATACCTCC |
| *BnPSP-1*-4 | ACTTCTCACCGAATAACGCAATCT |
| *BnPSP-1*-P1 | AAAGAACTGAGACAATGATGGTGA |
| *BnPSP-1*-P2 | TCTCTGTCTTTATTTTTCNNNNNNNNNN |
| *BnPSP-1*-P3 | TAAACTATTCCTCTGTGTCTTTCT |
| *BnPSP-1*-P4 | CTTTTTTTGGAGTTAGTGTTTTGA |
| *BnPSP-2*-1 | TGTTGTGTGGGGTTAGAGTTTGAGT |
| *BnPSP-2*-2 | TATTGAGTGGTAGAANNNNNNNNNN |
| *BnPSP-2*-3 | ATGGAGTGAATGACTGCGTTAGATG |
| *BnPSP-2*-4 | GTCGCCTCTTTGGGATATTGTTATG |
| *BnPSP-4*-1 | CGTGAGACAGATGCCCAATATAAC |
| *BnPSP-4*-2 | TGTGCTCAACTTGCGNNNNNNNNNN |
| *BnPSP-4*-3 | CAAATCCCCTAATGTGGCTCTTAAT |
| *BnPSP-4*-4 | GGAATATCGCTTCACTTTATGCTG |

“N” represents any nucleotide (A, T, C or G).

##### Supplementary Table 3. The primers information of promoter regions detection

| Name | Primer sequence(5´-3´) |
| --- | --- |
| *BnPSP-1*-F | AGTTGTGTATTGTTGCATCTTCCA |
| *BnPSP-1*-R | TCGTACAATTAACCCCAAAACCA |
| *Bnpsp-2*-F | AAAGGGAATTAGCGAGTGATGAAG |
| *Bnpsp-2*-R | ACTTCATCTAATTCCGAACAGACC |
| *BnPSP-4*-F | TACCTCAAAACAAGTCATATTCCCA |
| *BnPSP-4*-R | GGAGAGGATAGGGGAGCGA |
| M13-F | GTAAAACGACGGCCAGT |
| M13-R | CAGGAAACAGCTATGAC |

##### Supplementary Table 4. The primers for constructing binary expression vectors

| Name | Primer sequence(5’-3’) |
| --- | --- |
| *BnPSP-1*-1F | GACCATGATTACGCCAAGCTTCACTTTTGCACACCTAGACA |
| *BnPSP-1*-3F | GACCATGATTACGCCAAGCTTAATTTAATAACAAATAAGTC |
| *BnPSP-1*-4F | GACCATGATTACGCCAAGCTTCGAATATCATTAGCATTTAA |
| *BnPSP-1*-5F | GACCATGATTACGCCAAGCTTAGAGAGTGAAGAGAGAGAG |
| *BnPSP-1*-6F | GACCATGATTACGCCAAGCTTGTTTAAAAGACGAGGGCTTT |
| *BnPSP-1*-1R | GGACTGACCACCCGGGGATCCTTCTACAATTACCCAGAAA |
| *BnPSP-1*-2R | GGACTGACCACCCGGGGATCCTATTCCTCTGTGTCTTTCTC |
| *BnPSP-3*-1F | GACCATGATTACGCCAAGCTTACATTCCAGATCCTATATCG |
| *BnPSP-3*-2F | GACCATGATTACGCCAAGCTTTGTTGGAAGTGGAGTTAGAG |
| *BnPSP-3*-1R | GGACTGACCACCCGGGGATCCTGTTGCAACAACAACAATGT |
| *BnPSP-2*-1F | GACCATGATTACGCCAAGCTTTACCTCAAAACAAGTCATAT |
| *BnPSP-2*-5F | GACCATGATTACGCCAAGCTTCTATATAAACTCCCTCTCAC |
| *BnPSP-2*-1R | GGACTGACCACCCGGGGATCCTTCTTTTTTGCTAAATTTAG |
| VTF | CCCCAGGCTTTACACTTTATGCTT |
| VTR | CGCTGATCAATTCCACAGTTTTCG |

##### Supplementary Table 5. Putative cis-acting elements of PPSP1

| Name of cis-element | Sequence information | Position from translation start site | Function |
| --- | --- | --- | --- |
| GAREAT | TAACAAR | -1293 -751 | Gibberellin-responsive element |
| GARE1OSREP1 | TAACAGA | -896 | Gibberellin-responsive element |
| CURECORECR | GTAC | -641-1038-290 | Copper- and oxygen–responsive elements |
| ACGTATERD1 | ACGT | -1084 -210 | Induction by dehydration stress and dark-induced senescence |
| ARFAT | TGTCTC | -304 | ARF (auxin response factor) binding site |
| SURECOREATSULTR11 | GAGAC | -1365 | S responsive element |
| MYB1AT | WAACCA | -1084 -210 | ABA signal transduction |

##### Supplementary Table 6. Putative cis-acting elements of PPSP2

| Name of cis-element | Sequence information | Position from translation start site | Function |
| --- | --- | --- | --- |
| GARE1OSREP1 | TAACAGA | -359 | Gibberellin-responsive element |
| ABRELATERD1 | ACGTG | -970 | Early responsive to dehydration |
| CURECORECR | GTAC | -1596-1325  -684 -590 | Copper- and oxygen-responsive elements |
| ABRERATCAL | MACGYGB | -971 | Ca^2 +^ cis-acting element |
| DRE1COREZMRAB17 | ACCGAGA | -577 | ABA responsive element |
| SURE1STPAT21 | AATAGAAAA | -565 | Sucrose responsive element |
| LTRECOREATCOR15 | CCGAC | -225 | ABA responsiveness & necessary for cold- or drought- induced gene expression |
| GAREAT | TAACAAR | -2267 | Gibberellin-responsive element |
| -10PEHVPSBD | TATTCT | -1734 | Light-responsive element |
| SURECOREATSULTR11 | GAGAC | -1998 -1532 | S responsive element |

##### Supplementary Table 7. Putative cis-acting elements of PPSP4

| Name of cis-element | Sequence information | | Position from translation start site | Function |
| --- | --- | --- | --- | --- |
| ARFAT | | TGTCTC | -706 | ARF (auxin response factor) binding site |
| SURECOREATSULTR11 | | GAGAC | -1027 | S responsive element |
| SORLIP1AT | | GCCAC | -782-640 | Light-induced responsive element |
| ACGTATERD1 | | ACGT | -1503 -1359 -702  -655 -521 -471 -299 | Induction by dehydration stress and dark-induced senescence |
| AUXRETGA2GMGH3 | | TGACGTGGC | -523 -301 | IAA inducing element |
| CURECORECR | | GTAC | -2111 -1862 -1724  -1425 -1302 -1292  -1275 -700 -549 -194 | Copper- and oxygen–responsive elements |
| BOXIIPCCHS | | ACGTGGC | -521 -299 | Light-responsive element |
| ACGTABREMOTIFA2OSEM | | ACGTGKC | -521 -299 | ABA responsive element |
